# Chemerin in peritoneal sepsis and its associations with glucose metabolism and prognosis: a translational cross-sectional study

**DOI:** 10.1186/s13054-016-1209-5

**Published:** 2016-02-12

**Authors:** Paul Horn, Uta Barbara Metzing, Ricardo Steidl, Bernd Romeike, Falk Rauchfuß, Christoph Sponholz, Daniel Thomas-Rüddel, Katrin Ludewig, Andreas L. Birkenfeld, Utz Settmacher, Michael Bauer, Ralf Alexander Claus, Christian von Loeffelholz

**Affiliations:** Integrated Research and Treatment Centre, Centre for Sepsis Control and Care (CSCC), Jena University Hospital, Jena, Germany; Department of Anaesthesiology and Intensive Care Medicine, Jena University Hospital, Jena, Germany; Section of Neuropathology, Department of Pathology, Jena University Hospital, Jena, Germany; Department of General, Visceral and Vascular Surgery, Jena University Hospital, Jena, Germany; Section of Metabolic and Vascular Medicine, Medical Clinic III, University Hospital Carl Gustav Carus, Dresden, Germany; German Centre for Diabetes Research (DZD e.V.), Neuherberg, Germany; Section of Diabetes and Nutritional Sciences, Rayne Institute, King’s College London, London, UK

**Keywords:** Chemerin, Sepsis, Glucose homeostasis, Stress hyperglycaemia, Adipose tissue

## Abstract

**Background:**

Stress hyperglycaemia (SHG) is a common complication in sepsis associated with poor outcome. Chemerin is an adipocytokine associated with inflammation and impaired glucose homeostasis in metabolic diseases such as type 2 diabetes (T2D). We aimed to investigate how alterations of circulating chemerin levels and corresponding visceral adipose tissue (VAT) expression are linked to glucose metabolism and prognosis in sepsis.

**Methods:**

Clinical data and tissue samples were taken from a cross-sectional study including control, T2D and sepsis patients, all undergoing laparotomy. A second independent patient cohort of patients with sepsis was included to evaluate associations with prognosis. This was complemented by a murine model of peritoneal infection and a high-fat diet. We analysed circulating chemerin by enzyme-linked immunosorbent assay and VAT messenger RNA (mRNA) expression by real-time polymerase chain reaction.

**Results:**

Circulating chemerin was increased in sepsis 1.69-fold compared with controls (*p* = 0.012) and 1.47-fold compared with T2D (*p* = 0.03). Otherwise, chemerin VAT mRNA expression was decreased in patients with sepsis (*p* = 0.006) and in septic diabetic animals (*p* = 0.009). Circulating chemerin correlated significantly with intra-operative glucose (*r* = 0.662; *p* = 0.01) and in trend with fasting glucose (*r* = 0.528; *p* = 0.052). After adjusting for body mass index or haemoglobin A1c, chemerin correlated in trend with insulin resistance evaluated using the logarithmised homeostasis model assessment of insulin resistance (*r* = 0.539, *p* = 0.071; *r* = 0.553, *p* = 0.062). Chemerin was positively associated with Acute Physiology and Chronic Health Evaluation II score in patients with sepsis (*p* = 0.036) and with clinical severity in septic mice (*p* = 0.031). In an independent study population, we confirmed association of chemerin with glucose levels in multivariate linear regression analysis (β = 0.556, *p* = 0.013). In patients with sepsis with SHG, non-survivors had significantly lower chemerin levels than survivors (0.38-fold, *p* = 0.006), while in patients without SHG, non-survivors had higher chemerin levels, not reaching significance (1.64-fold, *p* = 0.089). No difference was apparent in patients with pre-existing T2D (*p* = 0.44).

**Conclusions:**

We show, for the first time to our knowledge, that chemerin is increased in sepsis and that it associates with impaired glucose metabolism and survival in these patients. It could be further evaluated as a biomarker to stratify mortality risk of patients with SHG.

**Electronic supplementary material:**

The online version of this article (doi:10.1186/s13054-016-1209-5) contains supplementary material, which is available to authorized users.

## Background

*Sepsis* is defined as a state of high-grade systemic inflammation caused by infection. This is accompanied by severe dysregulation of glucose homeostasis, including insulin resistance and stress hyperglycaemia (SHG) [1] as common complications associated with poor outcome [2] and post–intensive care unit (post-ICU) diabetes [3]. Besides glucocorticoid and catecholamine effects, insulin resistance is thought to be caused mainly by pro-inflammatory cytokines in sepsis [1]. These connections have been studied mainly in obesity-related diseases but are poorly characterised in sepsis. Deeper insights into these mechanisms may serve to reveal new therapeutic targets to control homeostasis as well as diagnostic and prognostic biomarkers of host response.

Adipokines have been receiving more attention in recent research in this area, as they are found to be related to inflammation, disturbed glucose metabolism and even disease severity in sepsis [4]. Chemerin (retinoic acid receptor responder 2 (RARRES2); NCBI Reference Sequence: [NM_002889]) is expressed mainly by adipose tissue [5]. Additional sites of expression include the liver, pancreas and adrenal gland [6, 7]. The inactive pro-chemerin is processed by serine and cysteine proteases, resulting in various chemerin isoforms with different activities and spatiotemporal distributions [8]. Known effects of chemerin include chemoattraction and activation of antigen-presenting cells, resulting in infiltration of leucocytes to different sites of inflammation and tissue damage [9] while retaining neutrophil granulocytes in the circulation [10, 11]. Systemic chemerin administration results in whole-body insulin resistance [12] and differentially regulates insulin sensitivity in different cell types, such as adipocytes [13] and muscle cells [14, 15]. Clear evidence exists as well for anti-inflammatory [10, 11, 16] and anti-microbial [17] properties of chemerin. Chemerin effects are mediated mainly by binding to its receptor chemokine-like receptor 1 (CMKLR1, also known as *ChemR23*) on CMKLR1^+^ cells such as leucocyte subpopulations, adipocytes and hepatocytes [6], as well as by endothelial activation [18]. C-C chemokine receptor-like 2 (CCRL2) and G protein–coupled receptor 1 (GPR1) are additional chemerin receptors responsible for modulating local chemerin activity [9]. Chemerin is thought to play a role in different metabolic, inflammatory and autoimmune processes such as metabolic syndrome [19], non-alcoholic fatty liver disease [20, 21], rheumatoid arthritis [22], chronic inflammatory bowel disease [23] and chronic pancreatitis [24].

On the basis of mechanisms of chemerin action, one can hypothesise that it is involved in differential leucocyte recruitment and regulation of inflammation, thereby modulating sepsis disease severity. At the same time, chemerin might participate in induction of insulin resistance and thus contribute to development of SHG. Thereby, chemerin is a possible link between systemic inflammation and insulin resistance in sepsis host response. However, chemerin has not been investigated in sepsis yet. Hence, we aimed to study chemerin in the context of sepsis host response in a clinical cross-sectional study with type 2 diabetes (T2D) as a positive control. We focused on changes in serum levels and their association with disturbed glucose metabolism, disease severity and prognosis. Furthermore, we wanted to evaluate the suitability of the murine peritoneal contamination and infection (PCI) model of peritoneal sepsis in combination with a T2D model for studying chemerin in the context of sepsis.

## Methods

### Study design

The main part of this study was a cross-sectional clinical analysis of chemerin and its associations with parameters of inflammation and disturbed glucose metabolism in patients with sepsis, T2D or none of the aforementioned diseases. We included patients with T2D as positive controls, in whom chemerin has already been described to be increased [25], and patients without metabolic co-morbidities or sepsis as negative control subjects. We took blood and visceral adipose tissue (VAT) samples from patients undergoing therapeutic laparotomy and collected data from standard laboratory measurements. We included blood samples and data from an independent cross-sectional study to validate associations with prognosis (validation cohort).

Clinical studies were complemented by a murine model of polymicrobial peritoneal sepsis. To accord with the study design of our clinical study, we included a murine diabetes model by feeding mice a high-fat diet (HFD).

### Clinical study subjects and ethics

Clinical tissue samples from the first study cohort were obtained from study subjects who were enrolled in the INSIGHT study, which is registered in the German Clinical Trials Register (DRKS00005450). As a second independent study population, we selected all consecutive patients from the HMOX study who had an abdominal focus of sepsis. The study approval was given by the faculty ethics review board of Jena University Hospital (INSIGHT: 3247-09/11 and validation cohort: 3624-11/12, 2712-12/09, 2160-11/07). All subjects or their legal representatives gave written informed consent and principal investigators of the original trials approved publication of this article. Inclusion and exclusion criteria for the two studies are provided in Additional file 1: Table S1. For intergroup comparison analyses, we included subgroups with ten patients each.

The diagnosis of sepsis was made following criteria of the S2k guidelines of the German Sepsis Society [26]. Patients with T2D fulfilled the 2010 criteria of the American Diabetes Association for a diagnosis of T2D [27] or had known T2D treated with oral anti-diabetic therapy. In addition to the exclusion criteria provided in Additional file 1: Table S1, control patients had no sepsis, T2D or metabolic syndrome as defined by National Cholesterol Education Program Expert Panel on Detection Evaluation, and Treatment of High Blood Cholesterol in Adults (Adult Treatment Panel III) criteria [28]. Alcohol abuse as an exclusion criterion was defined as a daily alcohol intake of more than 20 g for females and more than 40 g for males [29]. Each patient’s medical history was evaluated, and body mass index (BMI) was calculated for each patient. We calculated Acute Physiology and Chronic Health Evaluation II (APACHE II) score [30] and Simplified Acute Physiology Score II [31] as described elsewhere in the literature. In the second study cohort, we assigned patients to the newly diagnosed SHG subgroup on the basis of routine glucose measurements during the ICU stay according to predefined criteria [32].

### Human blood and tissue sampling

Control and T2D subjects of the first study cohort were fasted overnight, and peripheral blood was taken the morning before surgery. Blood from sepsis subjects was taken on the morning after surgery to obtain blood after an overnight fast, as a fasting period before surgery was not feasible. In the second, independent study cohort, blood was taken within 24 h of sepsis diagnosis. Serum was obtained by centrifugation and stored at −80 °C. VAT samples were taken right after mid-line incision and adhesiolysis from the greater omentum and immediately snap-frozen [33] in liquid nitrogen and stored at −80 °C. Blood samples of the second study cohort were taken on the morning of study enrolment.

To assess insulin resistance, we calculated the homeostasis model assessment of insulin resistance (HOMA-IR) [34] on the basis of fasting glucose and insulin levels. As an assessment of long-term glycaemia, we measured glycated haemoglobin A1c (HbA1c) by means of standard laboratory methods. All laboratory analyses were performed in certified clinical chemistry laboratories of Jena University Hospital.

### Confirmational exploration in an animal model

We performed all investigations and experiments in accordance with the German legislation on protection of animals and obtained permission to conduct the study from the regional animal welfare committee (registration number 02-038/12). Six-week-old male C57BL/6 J mice were kept under standardised laboratory conditions and were fed a standard rodent chow [control diet (CD)] or an HFD (EF R/M D12492 modified diet; ssniff, Soest, Germany) containing 34 % fat for 12 weeks. HFD served as a model of obesity-induced T2D [35] to reflect our clinical study design. We weighed the mice every 2 weeks to check for adequate feeding and weight development. Intra-peritoneal glucose tolerance tests were performed to verify diabetes-like phenotype. Therefore, the mice were given intra-peritoneal injections of 2 g/kg body weight glucose, and full blood glucose levels were measured at 0, 30, 60, 90 and 120 minutes after injection. Areas under the curve were calculated and compared between groups.

After 12 weeks of feeding, we randomised animals into four groups: CD baseline (CD_0h_), HFD baseline (HFD_0h_), CD sepsis (CD_24h_) and HFD sepsis (HFD_24h_). Sepsis was induced using the PCI model as described previously [36]. Briefly, a standardised and diluted suspension of human faecal slurry (1.25 μl/g body weight) was administered intra-peritoneally. Disease severity was assessed by using the Clinical Severity Score (CSS) as described elsewhere [36]. Twenty-four hours after sepsis induction, mice were killed while in deep isoflurane anaesthesia by taking citrate anti-coagulated blood by heart puncture. Plasma was made by centrifugation and stored at −80 °C. Epididymal fat pads were collected as VAT samples and immediately snap-frozen in liquid nitrogen and stored at −80 °C.

### Quantification of circulating chemerin

Circulating chemerin concentrations were measured from human serum and murine citrate anti-coagulated plasma. The enzyme-linked immunosorbent assays (ELISAs) used were the Human Chemerin ELISA kit (BioVendor, Kassel, Germany) and Mouse Chemerin Quantikine ELISA Kit (R&D Systems, Minneapolis, MN, USA). We performed ELISAs according to the manufacturer’s instructions, measured all samples in duplicates and calculated the means.

### VAT mRNA expression

We extracted VAT messenger RNA (mRNA) using the RNeasy Lipid Tissue Mini Kit (QIAGEN, Hilden, Germany) according to the manufacturer’s instructions. RNA concentration was measured by spectrophotometry with the NanoDrop 1000 instrument (NanoDrop Products/Thermo Scientific, Wilmington, DE, USA). The integrity of the RNA was checked by automated electrophoresis with the Experion Automated Electrophoresis System (Bio-Rad Laboratories, Hercules, CA, USA). Reverse transcription of RNA into complementary DNA (cDNA) was achieved by using the Fermentas RevertAid First Strand cDNA Synthesis Kit (Thermo Scientific, Grand Island, NY, USA) according to the manufacturer’s instructions. Real-time quantitative polymerase chain reactions (RT-qPCR) were performed using the Rotor-Gene Q cycler (QIAGEN).

For RT-qPCR analysis, we used the primers listed in Additional file 1: Table S2. Relative expression for each primer was interpolated from standard curves. After housekeeping gene analysis using geNorm [37] and NormFinder [38] software, the means of relative expression of β actin, glyceraldehyde 3-phosphate dehydrogenase (GAPDH), hypoxanthine phosphoribosyltransferase 1 (HPRT) and porphobilinogen deaminase (PBGD) were calculated and used as normalisation factors in clinical studies and Gapdh and Hprt in mouse studies. Normalised gene expression is given as relative expression of the gene of interest divided by the normalisation factor for each sample.

### Statistical analysis

IBM SPSS Statistics version 22.0 software (IBM, Armonk, NY, USA) was used to perform statistical analyses. An alternative hypothesis was accepted as a two-sided *p* < 0.05. All data are given as mean ± standard error of the mean if not stated otherwise. Normal distribution was tested with the Shapiro-Wilk test, and decade logarithmic transformation was used if required. To test for homogeneity of variance, we used Levene’s test. Depending on data distribution, we used the following statistical procedures: one-way analysis of variance with post hoc Bonferroni adjustment, Kruskal-Wallis test or Mann-Whitney *U* test. To test for correlation, Pearson’s simple correlation coefficient or Spearman’s rank correlation coefficient was applied. Multivariate linear regression analysis was applied with circulating chemerin levels as the dependent variable to test for independent linear associations.

## Results

### Subject characterization

The characteristics of patients in the first study cohort are provided in Table 1. The groups were different in terms of age (*p* < 0.001), BMI (*p* = 0.003), HbA1c (*p* < 0.001) and HOMA-IR (*p* = 0.022). Patients with sepsis were comparable to subjects with T2D with respect to age (*p* = 0.80), BMI (*p* = 0.52) and HOMA-IR (*p* = 0.44) and comparable to control subjects with respect to HbA1c (*p* = 0.13). Indications for surgery were not different between control and T2D subjects (Additional file 1: Table S4), with a higher number of primary hepatic or biliary malignancies in patients with T2D. Patients in the sepsis group underwent emergency or high urgent surgery for different indications. T2D and sepsis patients were matched according to age and BMI to make these groups of our main interest more comparable for subgroup comparison analyses. The patient characteristics of these subgroups are provided in Additional file 1: Table S3.

**Table 1:** Characteristics of the first study cohort

	Control	T2D	Sepsis	*p* Value
Number of subjects	17	20	14	–
Male sex, %	30	80	57	0.008^a^
Age, yr	56 ± 3^b^	68 ± 1^c^	69 ± 3^c^	<0.001
BMI, kg/m^2^	24.8 ± 1.0^b^	28.9 ± 0.9^c^	29.5 ± 1.2^c^	0.003
HbA1c, %	5.5 ± 0.1^b^	7.8 ± 0.4^c^	5.9 ± 0.3^b^	<0.001
HOMA-IR, AU	1.5 ± 0.3^b^	5.6 ± 1.4^c^	4.3 ± 1.3^c^	0.022
GFR, ml/min	95.3 ± 3.0^b^	70.8 ± 3.7^c^	44.9 ± 10.5	<0.001
CRP, mg/L	3.5 ± 0.5^b^	15.9 ± 9.5^c^	220.5 ± 26.8^d^	<0.001
Malignancy, %	65	80	64	0.736^a^
Duration of ICU stay, days			7.8 ± 2.1	–
ICU non-survivors, n			5/14	–

*Abbreviations*: *BMI* body mass index, *CRP* C-reactive protein, *GFR* glomerular filtration rate, *HbA1c* haemoglobin A1c, *HOMA-IR* homeostasis model assessment of insulin resistance, *ICU* intensive care unit, *T2D* type 2 diabetes mellitus

Data are given as mean ± SEM, absolute numbers or median and interquartile range. For comparison of groups, the Kruskal-Wallis test and post hoc Bonferroni adjustment were used. Superscript letters indicate significant differences between subgroups (*p*<0.05)

^a^χ^2^ test

In a second independent study cohort, we included samples of 37 patients with peritoneal sepsis. We categorised these patients into three groups according to their glycaemic status during their ICU stay: patients without SHG, patients with previously diagnosed T2D and patients with SHG but without a history of diabetes mellitus. The characteristics of the second study cohort are shown in Table 2. Groups were different in terms of age and BMI, with significantly younger and leaner patients in the SHG group.

**Table 2:** Characteristics of the validation cohort

		Glycaemic status on ICU			
	All	No SHG	T2D	New SHG	*p* Value
Number of subjects	37	11	11	15	
Male sex, %	65	64	64	67	0.982^a^
Age, yr	63 ± 2	68 ± 3^b^	70 ± 2^b^	54 ± 4^c^	0.002
BMI, kg/m^2^	27.8 ± 1.0	28.0 ± 2.2^b^	31.2 ± 2.0^b^	25.2 ± 1.1^b^	0.064
Glucose mmol/L	6.8 ± 0.3	6.6 ± 0.3	6.5 ± 0.6	7.2 ± 0.4	0.264
GFR ml/min	62.4 ± 6.9	62.3 ± 11.0^b^	39.8 ± 8.0^b^	80.4 ± 12.8^b^	0.033
CRP mg/L	142.0 ± 18.8	149.4 ± 39.3^b^	170.7 ± 37.4^b^	115.6 ± 95.8^b^	0.477
Diabetes mellitus	12/37	0/11	11/11	0/15	<0.001^a^
Renal replacement therapy	3/37	1/11	1/11	1/15	0.965^a^
APACHE II score	21 (18–28)	20^b^ (18–25)	27^b^ (20–33)	21^b^ (15–26)	0.185
SAPS II	49 (37.0–64.5)	48^b^ (42.0–65.0)	63^b^ (48.0–71.0)	45^b^ (31.0–54.0)	0.112
28-day mortality, n (%)	10/37 (27)	4/11 (36)	2/11 (18)	4/15 (27)	0.628^a^

*Abbreviations*: *APACHE II* Acute Physiology and Chronic Health Evaluation, *BMI* body mass index, *CRP* C-reactive protein, *GFR* glomerular filtration rate, *ICU* intensive care unit, *SAPS II* Simplified Acute Physiology Score, *SHG* stress hyperglycaemia, *T2D* type 2 diabetes

Data are given as mean ± standard error of the mean, absolute numbers or median and interquartile range. For comparison of groups, the Kruskal-Wallis test and post hoc Bonferroni adjustment were used. Superscript letters indicate significant differences between subgroups (*p*<0.05)

^a^χ^2^ test

In mice, HFD induced substantial and significant weight gain compared with the CD (Additional file 2: Fig. S1A). In accordance with a diabetes-like phenotype of HFD-fed mice, impaired glucose tolerance and higher baseline glucose levels were observed (Additional file 2: Fig. S1).

### Circulating chemerin and mRNA VAT expression

**Fig. 1:**
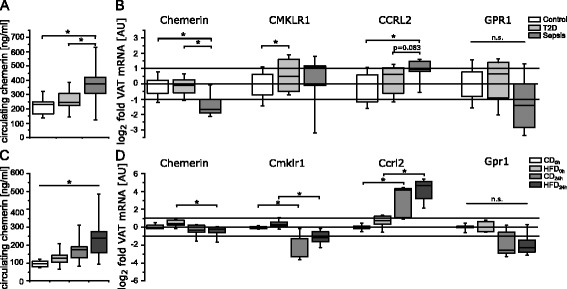
Circulating levels and visceral adipose tissue mRNA expression of chemerin. One-way analysis of variance with Bonferroni adjustment, Kruskal-Wallis test or Mann-Whitney *U* test with Bonferroni-Holm adjustment were applied (*p<0.05). **a** Circulating chemerin levels in matched clinical study subjects. **b** mRNA expression of chemerin and its receptors in VAT of matched clinical study subjects. **c** Circulating chemerin levels in the mouse model of diabetes and sepsis (n = 5 or 6 per group). **d** mRNA expression of chemerin and its receptors in VAT of diabetic and septic mice (n = 5 or 6 per group). *VAT* visceral adipose tissue, *AU* arbitrary units, *CCRL2* C-C chemokine receptor-like 2, *CMKLR1* chemokine-like receptor 1, *GPR1* G protein–coupled receptor 1, *T2D* type 2 diabetes, *mRNA* messenger RNA, *n.s.* not significant, *CD*_*0h*_ control diet baseline, *HFD*_*0h*_ high-fat diet baseline, *CD*_*24h*_ control diet sepsis, *HFD*_*24h*_ high-fat diet sepsis

Circulating chemerin levels in human sepsis were significantly increased by 1.69-fold compared with controls (*p* = 0.012) and by 1.47-fold compared with T2D (*p* = 0.03) (Fig. 1a). No significant difference was observed between controls and T2D (*p* = 0.87) (Fig. 1a). In patients with sepsis, we observed a strong positive correlation of chemerin with blood leucocyte count (*p* = 0.02) and thrombocyte count (*p* = 0.03), but not with C-reactive protein (CRP), interleukin-6 or glomerular filtration rate (Additional file 1: Table S5). No such significant correlations were found in the validation cohort (Additional file 1: Table S6). Other associations of chemerin with clinical characteristics are shown in Additional file 1: Table S5 and Table S6.

VAT mRNA expression of chemerin was markedly lower in patients with sepsis compared with T2D (*p* = 0.012) and controls (*p* = 0.009) (Fig. 1b). No difference between controls and T2D was apparent (*p* = 0.80). Expression of CMKLR1 was increased in patients with T2D compared with controls (*p* = 0.029) but was not altered in sepsis (Fig. 1b), while CCRL2 mRNA was increased in patients with sepsis compared with controls (*p* = 0.031; Fig. 1b) and no significant differences among groups were observed for GPR1 (Fig. 1b).

In concordance with the clinical results, circulating chemerin levels were significantly different across all mouse groups (*p* = 0.003), with the highest levels in septic mice (Fig. 1c). Murine chemerin mRNA levels in VAT were reduced in HFD_24h_ and CD_24h_ undergoing sepsis compared with HFD_0h_ (*p* = 0.009 and *p* = 0.012, respectively) but not CD_0h_ (*p* = 0.53 and *p* = 0.75, respectively) (Fig. 1d). Cmklr1 mRNA was significantly decreased in both CD and HFD mice following sepsis (*p* = 0.001 and *p* < 0.001, respectively) (Fig. 1d), while Ccrl2 mRNA was increased (*p* = 0.024 and *p* = 0.012, respectively) (Fig. 1d). Murine Gpr1 mRNA was not significantly different among groups (Fig. 1d).

### Association with parameters of glucose metabolism

**Fig. 2:**
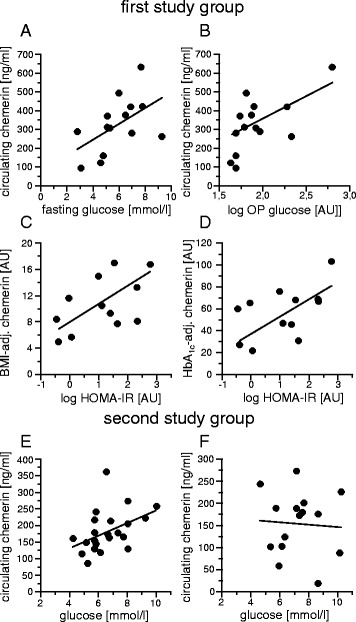
Association of chemerin with markers of disturbed glucose homeostasis. Pearson’s simple coefficient or Spearman’s rank correlation coefficient was used for correlation analyses. **a** Correlation of circulating chemerin levels with fasting glucose levels (*r* = 0.528, *p* = 0.052, *n* = 14). **b** Correlation of circulating chemerin levels with logarithmised intraoperative (OP) glucose in subjects with sepsis (*r* = 0.662, *p* = 0.01, *n* = 14). **c** Correlation of BMI-adjusted circulating chemerin levels with HOMA-IR (*r* = 0.539, *p* = 0.071, *n* = 12). **d** Correlation of HbA1c-adjusted circulating chemerin levels with HOMA-IR after adjusting for BMI (*r* = 0.553, *p* = 0.062, n = 12). **e** Correlation of circulating chemerin levels with glucose levels in the second study cohort after exclusion of patients with SHG (*r* = 0.438, *p* = 0.041, *n* = 22). **f** Correlation of circulating chemerin levels with glucose levels in patients with SHG in the second study cohort (*r* = −0.063; *p* = 0.823, *n* = 15). *AU* arbitrary units, *BMI* body mass index, *HbA1c* haemoglobin A1c, *HOMA-IR* homeostasis model assessment of insulin resistance, *SHG* stress hyperglycaemia

In patients with sepsis, circulating chemerin correlated in trend with fasting glucose levels (*r* = 0.528, *p* = 0.052) (Fig. 2a) and significantly with logarithmised intra-operative glucose levels (*r* = 0.662, *p* = 0.01) (Fig. 2b). In control patients, we found comparable significant positive correlations of chemerin with intra-operative glucose (*r* = 0.549, *p* = 0.023) (Additional file 3: Fig. S2) and fasting glucose levels (*r* = 0.516, *p* = 0.034) (Additional file 3: Fig. S2), while no significant correlations were found in patients with T2D (Additional file 4: Fig. S3). After adjusting circulating chemerin levels by dividing by BMI or HbA1c, chemerin correlated with logarithmised HOMA-IR, but without reaching significance (*r* = 0.539, *p* = 0.071, and *r* = 0.553, *p* = 0.062, respectively) (Fig. 2c and d). Because two values of HOMA-IR equalled 0, those values dropped out for logarithmised analysis. In control subjects and patients with T2D, no correlation of chemerin and HOMA-IR was apparent (data not shown).

In the second independent study cohort, we did not observe a significant association of chemerin with glucose levels (Additional file 1: Table S6). However, after exclusion of patients with SHG, chemerin was positively and significantly correlated to glucose levels (*r* = 0.438, *p* = 0.041) (Fig. 2e), while no correlation was observable in SHG (*r* = −0.063, *p* = 0.82) (Fig. 2f). In a multivariate linear regression model, circulating chemerin was significantly and independently associated with glucose levels in patients without SHG (β = 0.556, *p* = 0.013) (Model Sepsis C; see Table 3).

**Table 3:** Multivariate linear regression analysis with blood glucose

	Model Sepsis C
	Dependent variable: blood glucose
	β coefficient (*p* value)
Independent variables	*R* = 0.800; *R* ^2^ = 0.640; adjusted *R* ^2^ = 0.431, *p* = 0.043
Sex	−0.023 (0.920)
Age	0.118 (0.549)
Log BMI	−0.506 (0.096)
Circulating chemerin	0.556 (0.013)
Blood Urea	−0.773 (0.004)
Log bilirubin	0.104 (0.618)
Log lactate	−0.449 (0.048)

*BMI* body mass index

### Association with and disease severity

**Fig. 3:**
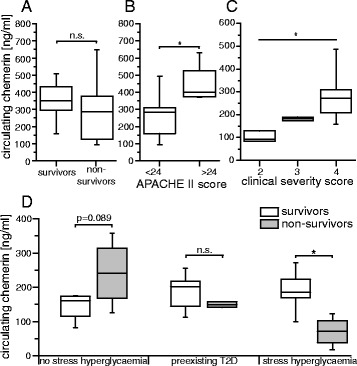
Circulating chemerin and markers of prognosis and disease severity. The Mann-Whitney *U* test or the Kruskal-Wallis test was applied (*p*<0.05). **a** Circulating chemerin was not significantly different between survivors and non-survivors of sepsis in the first study group. **b** Elevated circulating chemerin in sepsis subjects with an APACHE II score higher than 24 (*p* = 0.036). **c** Increased circulating chemerin levels with higher Clinical Severity Score in septic mice (*n* = 2 for scores 2 and 3, *n* = 7 for score 4). **d** Chemerin levels, depending on survival status of patients with sepsis without stress hyperglycaemia (SHG), with pre-existing type 2 diabetes mellitus or with SHG in the second study group. *APACHE II* Acute Physiology and Chronic Health Evaluation

In the primary study group, chemerin was not associated with 28-day survival after sepsis onset (Fig. 3a), but we found increased chemerin levels in patients with an APACHE II score higher than 24 points at study enrolment (*p* = 0.036) (Fig. 3b) [30]. In concordance with these results, an increased CSS [36], as an assessment of sepsis disease severity in rodents, was significantly accompanied by higher levels of circulating chemerin in septic mice (*p* = 0.031) (Fig. 3c).

In the next step, we aimed to evaluate prognostic associations of chemerin in our second independent study cohort. In patients with SHG, survivors displayed significantly higher chemerin levels than non-survivors (*p* = 0.006) (Fig. 3d). In contrast, survivors displayed lower chemerin levels than non-survivors in patients without SHG, not reaching significance (*p* = 0.089) (Fig. 3d). No such association was visible in patients with sepsis with pre-existing T2D (*p* = 0.436) (Fig. 3d).

Excluding patients with T2D for further analyses, we retrospectively defined two risk groups: (1) patients without SHG and chemerin levels higher than 200 ng/ml and (2) patients with SHG and chemerin levels lower than 100 ng/ml. Assigning patients retrospectively into one of those groups resulted in a sensitivity of 75 % and a specificity of 89.5 % for detecting death at day 28 after sepsis onset. The positive predictive value for this population was 85.7 %, and the negative predictive value was 94.4 %. Patients assigned to one of the two risk groups had an 8.1-fold incidence of death during the first 28 days of sepsis (95 % confidence interval 2.1–31.5, *p* < 0.05).

## Discussion

In our cross-sectional study, we found increased circulating chemerin levels and reduced VAT chemerin mRNA in patients with peritoneal sepsis. In addition, we found associations of chemerin with glucose metabolism, disease severity and prognosis. The major findings of the clinical study were supported in a murine model of sepsis and diabetes.

As a distinct advantage, we provide highly characterised study subjects and adipose tissue gene expression data. Due to the study design with the need for VAT samples and thus an indication for open abdominal surgery, our number of study subjects was limited. However, our study yielded significant differences supported by our animal model, indicating sufficient power and validity. Our study subjects in the primary study cohort were representative of common surgical patients undergoing abdominal surgery, including a high incidence of malignant disease. By enrolling patients with T2D as intended positive controls, we were able to compare patients with sepsis with high-grade inflammation and acute disturbance of glucose metabolism with those who had low-grade inflammation and chronic disturbance of glucose metabolism. This is represented by high CRP, low HbA1c and high HOMA-IR in sepsis and lower CRP, high HbA1c and high HOMA-IR in T2D. Age and obesity are risk factors for both development of T2D [27] and sepsis [39], which is why both study groups were different from controls with regard to age and BMI. We intended to include patients with T2D as positive controls, referring to previous reports of increased chemerin levels in T2D [25]. However, we failed to reproduce these results, most likely due to co-morbidities in the control group, as chemerin levels in healthy control subjects are reported to be lower than we describe [7]. Indications for surgery were comparable between controls and patients with T2D, while indications were substantially different in patients with sepsis, due to the nature of the disease.

Our study is the first, to our knowledge, to demonstrate increased circulating chemerin levels in sepsis host response. This increase exceeds elevated values, as found in other diseases with either low- or high-grade inflammation as a substantial component such as metabolic syndrome [5] and T2D [40], inflammatory bowel disease [23], chronic pancreatitis [24] and rheumatoid arthritis [41]. Chemerin values in the control group exceeded normal values described in young healthy women, ranging from 160 ng/ml in the fed state to 55 ng/ml in the fasted state [7]. Therefore, our concept of selecting T2D as a positive control condition failed.

Epitope recognition of available ELISAs is not suitable for differentiating distinct chemerin isoforms and is therefore unable to distinguish unprocessed, inactive prochemerin- and proteolysis-activated chemerin. Hence, it can be hypothesised whether increased chemerin levels in sepsis correspond to increased activity. An activation of chemerin in sepsis host response is likely, as it is activated by serine and cysteine proteases of neutrophil granules and the coagulatory cascade [8], both of which are activated in sepsis [42, 43]. As our independent study cohort was not designed to reproduce results of increased chemerin, an appropriate non-septic control group is lacking. However, data regarding increased chemerin in sepsis are supported by our mouse model of sepsis and diabetes, as we observed a stepwise increase of chemerin levels with metabolic disease induced by HFD, high-grade inflammation induced by PCI and the combination of both. Because in the literature both glucose homeostasis and inflammation are associated with chemerin levels [9–15], we interpret our results as a kind of a dose-dependent effect on circulating chemerin.

We analysed chemerin mRNA expression in VAT, as it is described as a main site of chemerin production [5]. Surprisingly, we found decreased chemerin mRNA expression in sepsis, despite its increased circulating levels. Chemerin mRNA has been shown to be positively regulated by peroxisome proliferator-activated receptor γ [44], which in turn is downregulated in response to inflammation in adipocytes [45]. Likewise, chemerin mRNA is upregulated by insulin [44], but insulin signalling may be disrupted in adipose tissue due to insulin resistance. However, chemerin secretion is not necessarily related to chemerin mRNA levels [46], whereby decreased chemerin mRNA expression may not necessarily result in decreased chemerin secretion by VAT. Our PCR results may be biased because we measured mRNA levels only in whole-tissue homogenates, but adipose tissue is comprised of many different cell types. Endothelial activation is shown to increase chemerin mRNA expression in endothelial cells [18], an effect that may have been obscured by mRNA downregulation in other cell types. Other possible sources for increased chemerin levels in the circulation are secretion from liver, pancreas or adrenal glands [7, 20, 40], but those organs were beyond the scope of our explorative study. Another explanation for increased levels of circulating chemerin levels would be changes in receptor expression. Absent CMKLR1 or CCRL2 protein in knockout mice is associated with increased chemerin levels [47, 48]. We did observe a substantial downregulation of CMKLR1 in our mouse model, but not in human VAT. Thereby decreased CMKLR1 expression could at least in part contribute to increased chemerin levels. We observed increased CCRL2 mRNA expression levels in human and murine VAT, which is described to happen in response to endothelial activation [48]. Thus, increased CCRL2 expression is unlikely to contribute to increased chemerin levels. Changes in GPR1 mRNA are unlikely to contribute, as knockout of GPR1 in mice does not change chemerin concentration [49] and we did not observe changed GPR1 expression.

The literature strongly suggests a role for inflammation and impaired renal function in elevation of chemerin levels in sepsis [9, 50]. While we did not observe consistent significant associations with inflammation or renal function in patients with sepsis, the T2D and sepsis groups in the primary study cohort were comparable in means of age, sex, BMI and HOMA-IR. As the increase in sepsis patients therefore cannot be completely attributed to altered glucose homeostasis, we assume that inflammation as well as impaired renal function may be responsible as additional influencing factors.

In both study cohorts, chemerin was clearly associated with parameters of disturbed glucose homeostasis and insulin resistance, excluding patients with SHG. It is well known that chemerin is associated with BMI [5], hyperinsulinemia [51], insulin resistance [52, 53] and dyslipidaemia [54] in diseases associated with development of the metabolic syndrome, such as T2D. Chemerin induces insulin resistance in muscle cells [14, 15] and promotes insulin sensitivity in adipocytes [13] in vitro. In vivo administration [12] or adenoviral overexpression of chemerin [15] promotes glucose intolerance and insulin resistance. Thereby increased circulating chemerin is likely to contribute to disturbed glucose homeostasis, insulin resistance and development of SHG in sepsis. Interactions of chemerin with other adipokines might be involved in regulation of glucose homeostasis [4], but they were beyond the scope of our study.

Furthermore, we evaluated associations of chemerin with disease severity and 28-day mortality. Chemerin was associated with the APACHE II score in the clinical study and with disease severity in the mouse model, implying chemerin as being of prognostic value. In our primary study group, we could not observe differences in chemerin levels between survivors and non-survivors. As the number of studied patients was rather small, a separate analysis of patients with SHG was not feasible. We included a second independent study cohort comprised of patients with abdominal sepsis to further investigate the association of chemerin with prognosis. Interestingly, we found inverse associations of chemerin with 28-day mortality, depending on glycaemic status. As the number of patients was very small for each group, our data have to be interpreted carefully. Also, it must be noted that patients with SHG were significantly younger and leaner than patients without SHG, which may reflect different underlying diseases. Additionally, chemerin failed to associate with glucose levels in patients with SHG, indicating different functional mechanisms of chemerin in this group. Though we did not investigate chemerin isoforms, differential chemerin processing may be a possible explanatory approach for this difference, as different isoforms have opposite functions in regulation of inflammation [44].

Novel data of chemerin function support the concept that chemerin is not a pro- or anti-inflammatory agent per se, but initiates infiltration of immunomodulatory CMKLR1^+^ cells [44], which then can initiate pro- or anti-inflammatory reactions, depending on other factors in the local milieu. An adaptive function of chemerin is supported by previous findings of direct anti-microbial effects [17, 55], and protective effects against lung inflammation and zymosan induced peritonitis [10, 11, 16]. In patients without SHG, chemerin was associated mainly with glucose levels. Adverse prognostic associations in this group thereby might be mediated by adverse effects of higher glucose levels [2]. Prospective studies are needed to confirm prognostic associations of chemerin, taking into account glycaemic status of patients and chemerin isoforms. Comparative studies in critically ill patients without sepsis are needed to provide evidence for whether these associations are specific for sepsis or generalisable for critical illness.

The PCI model of sepsis reflects the main characteristics of human sepsis [36]. The main findings of our clinical studies regarding circulating chemerin, its VAT gene expression and associations with disease severity were reproducible in the mouse model, but substantial differences between human and murine chemerin have been reported. Circulating chemerin levels show day–night variations in mice [46] but not in humans [7]. Chemerin mRNA expression patterns are reported to be different, with high adipose tissue expression in mice [56] and low adipose tissue expression in humans [7]; however, open access transcriptomic data indicate moderate to high chemerin expression in adipose tissue in humans as well [57]. Regarding the dependency of chemerin on the glycaemic status of patients with sepsis, no reliable animal models for SHG are available so far. Besides all its limitations, the PCI model of peritoneal sepsis and the HFD model of obesity-induced T2D seem to be suitable for studying chemerin function in sepsis host response in an appropriate manner.

## Conclusions

To the best of our knowledge, this report is the first to describe altered circulating chemerin levels in sepsis. Chemerin was found to be linked to disturbed glucose metabolism and may play a differential role in the pathogenesis of SHG. Additionally, we found chemerin to be associated with disease severity. It may even be suitable as a prognostic factor, depending on the glycaemic status of patients with sepsis. Our results imply differential mechanistic functions in patients with SHG. Our main clinical results were reflected in the PCI model of sepsis, which makes it suitable for studying mechanisms of chemerin action in sepsis host response.

In future studies, it will be important to examine different chemerin isoforms and their distribution to these effects as well as the underlying mechanisms. Prospective clinical trials are needed to confirm the prognostic value of chemerin in sepsis.

## Key messages

- Circulating chemerin levels are increased in peritoneal sepsis compared with control and T2D, while adipose tissue mRNA expression is decreased.
- The murine PCI model of peritoneal sepsis reliably reflects changes of circulating chemerin and adipose tissue mRNA expression in humans.
- Circulating chemerin levels are associated with disturbed glucose homeostasis and insulin resistance in sepsis.
- Circulating chemerin levels seem to be associated with prognosis, depending on glycaemic status.

## Additional files

Additional file 1:
**Table S1**: Inclusion and exclusion criteria for clinical studies. **Table S2**: Primer sequences and NCBI accession numbers. **Table S3**: Characterisitcs of matched subgroups. **Table S4**: Indications for open abdominal surgery. **Table S5**: Correlation of chemerin with clinical and paraclinical parameters in the first study cohort. **Table S6**: Correlation of chemerin with clinical and paraclinical parameters in second cohort. Additional file 2:
**Figure S1**. Characterisation of the murine HFD model. **a** Development of body weight over 12 weeks of high-fat feeding. HFD mice gained significantly more body weight (*n* = 15/group). **b** Baseline plasma glucose levels were significantly higher in HFD-fed mice after 12 weeks compared with CD-fed mice (*p* = 0.01, *n* = 6). **c** Intra-peritoneal glucose tolerance test (ipGTT) showed no significant difference in glucose tolerance after 4 weeks of HFD (*n* = 4 or 5). **d** Area under the curve (AUC) was not significantly different for ipGTT between HFD- and CD-fed mice after 4 weeks of feeding (*p* = 0.286; *n* = 4 or 5). **e** HFD-fed mice had decreased glucose tolerance in ipGTT after 8 weeks of feeding (*p* = 0.04; *n* = 5). **f** Area under the curve (AUC) was significantly higher in HFD-fed mice compared with CD-fed mice after 8 weeks of feeding (*p* = 0.008, *n* = 5). (PDF 47 kb) Additional file 3:
**Figure S2**. Correlation of fasting and intra-operative glucose levels with circulating chemerin in controls. **a** Correlation of circulating chemerin levels with fasting glucose levels (*r* = 0.216, *p* = 0.034, *n* = 17). **b** Correlation of circulating chemerin levels with OP glucose (*r* = 0.549, *p* = 0.023, *n* = 17). (PDF 44 kb) Additional file 4:
**Figure S3**. Correlation of fasting and intra-operative glucose levels with circulating chemerin in T2D. **a** Correlation of circulating chemerin levels with fasting glucose levels (*r* = −0.149, *p* = 0.53, *n* = 21). **b** Correlation of circulating chemerin levels with OP glucose (*r* = −0.025, *p* = 0.92, *n* = 21). (PDF 44 kb)

## Abbreviations

APACHE: Acute Physiology and Chronic Health Evaluation
AU: arbitrary units
BMI: body mass index
CCRL2: Ccrl2, C-C chemokine receptor-like 2
CD: control diet
cDNA: complementary DNA
CMKLR1: Cmklr1, chemokine-like receptor 1
CRP: C-reactive protein
CSS: Clinical Severity Score
ELISA: enzyme-linked immunosorbent assay
GAPDH: Gapdh, glyceraldehyde 3-phosphate dehydrogenase
GFR: glomerular filtration rate
GPR1: Gpr1, G protein–coupled receptor 1
HbA1c: haemoglobin A1c
HFD: high-fat diet
HOMA-IR: homeostasis model assessment of insulin resistance
HPRT: Hprt, hypoxanthine phosphoribosyltransferase 1
ICU: intensive care unit
ipGTT: intra-peritoneal glucose tolerance test
IQR: interquartile range
mRNAOP: messenger RNAintraoperative
PCI: peritoneal contamination and infection
RARRES2: Rarres2, retinoic acid receptor responder 2
RT-qPCR: real-time quantitative polymerase chain reaction
SAPS: Simplified Acute Physiology Score
SHG: stress hyperglycaemia
T2D: type 2 diabetes
VAT: visceral adipose tissue
