# Supplementary material for: Chemerin in peritoneal sepsis and its associations with glucose metabolism and prognosis: a translational cross-sectional study
Source: Crit Care. 2016 Feb 12;20:39. doi: 10.1186/s13054-016-1209-5 (PMC4751629; doi:10.1186/s13054-016-1209-5)
Supplement: Additional file 1: — Table S1: Inclusion and exclusion criteria for clinical studies. Table S2: Primer sequences and NCBI accession numbers. Table S3: Characterisitcs of matched subgroups. Table S4: Indications for open abdominal surgery. Table S5: Correlation of chemerin with clinical and paraclinical parameters in the first study cohort. Table S6: Correlation of chemerin with clinical and paraclinical parameters in second cohort. [file 13054_2016_1209_MOESM1_ESM.docx]

| **Table S1 Inclusion and exclusion criteria for clinical studies** | | |
| --- | --- | --- |
|  | **INSIGHT (first cohort)** | **validation cohort** |
| Inclusion criteria | - Age > 18 years - Indication for open abdominal surgery   Sepsis:   - Diagnosis of sepsis according to established criteria   T2D:   - Diagnosis of type 2 diabetes according to ADA criteria   Control:   - No diagnosis of sepsis, diabetes or metabolic syndrome | - Age > 18 years - Diagnosis of sepsis according to established criteria - Abdominal focus |
| Exclusion criteria | - Previous abdominal surgery in the last 5 days - chemotherapy within the last 2 months - long-term immune-suppressive medication - status after organ transplantation - chronic or acute active rheumatoid inflammatory disease - drug or alcohol abuse [[28](#_ENREF_28)] - pre-existing essential haemodialysis - liver cirrhosis. | None |

| **Table S2 Primer sequences and NCBI accession numbers** | | |
| --- | --- | --- |
| **gene symbol** | **5´ - 3´ primer sequence** | **NCBI accession number** |
| ACTB (hum) | fw: ggcatgggtcagaaggat t  rv: aggtgtggtgccagattttc | NM_001101.3 |
| GAPDH (hum) | fw: ctctgctcctcctgttcgac  rv: caa tac gaccaaatc cg ttg ac | NM_002046.5 |
| HPRT (hum) | fw: cctggcgtc gtg attagt gat  rv: agacgttcagtcctgtccata a | NM_000194.2 |
| HMBS(hum) | fw: atgtctggtaacggcaatgc  rv: cgtctg tat gcgagcaag c | NM_000190.3 |
| RARRES2 (hum) | fw: agacaagctgccggaaga gg  rv: tggagaaggcga act gtc ca | NM_002889.3 |
| Actb (mur) | fw: gctcttttc cag ccttcctt  rv: cggatgtcaacgtcacactt | NM_007393.4 |
| Gapdh (mur) | fw: caa cag caactcccactcttc  rv: ggtccagggtttctt act cct t | NM_001289726.1 |
| Gusb (mur) | fw: gaaacccgccgc ata tta c  rv: ccc cag gtctgcatcatatt | NM_010368.1 |
| Hmbs (mur) | fw: gaaatcattgctatgtccacc a  rv: gcgttttctagctccttggta a | NM_013551.2 |
| Rarres2 (mur) | fw: agacca act gccccaaga ag  rv: atttccgccttctcccgttt | NM_027852.2 |

ACTB, Actb: betaactin; GAPDH, Gapdh: glyceraldehyde-3-phosphate dehydrogenase; Gusb: betaglucuronidase; HPRT: hypoxanthinephosphoribosyltransferase 1;HMBS, Hmbs: hydroxymethylbilanesynthase; RARRES2, Rarres2: retinoicacidreceptorresponder 2.

| **Table S3 Characteristics of matched subgroups** | | | | |
| --- | --- | --- | --- | --- |
|  | **Control** | **T2D** | **Sepsis** | **p-value** |
| No. (% male) | **10** (30) | **10** (90) | **10** (80) | 0.01* |
| Age [years] | **61**±2^a^ | **67**±2^b^ | **66**±2^b^ | 0.094 |
| BMI [kg/m²] | **24.4**±0.9^a^ | **27.9**±0.6^b^ | **29.0**±1.6^b^ | 0.024 |
| HbA_1c_[%] | **5.5**±0.1^a^ | **8.2**±0.6^b^ | **5.7**±0.3^a^ | <0.002 |
| HOMA-IR [AU] | **1.4**±0.3^a^ | **7.5**±2.4^b^ | **5.0**±1.7^b^ | 0.089 |
| GFR [ml/min] | **90.3**±3.0 | **73.3**±5.2 | **46.4**±13.7 | 0.069 |
| CRP [mg/l] | **3.4**±0.7^a^ | **8.7**±2.1^b^ | **221.1**±33.3^c^ | <0.001 |
| Malignancy (%) | **70** | **80** | **70** | 0.861* |
| Duration of ICU stay | - | - | 6.2±1.6 | - |
| ICU non-survivors (%) | - | - | 4/10 | - |

Data are given as mean ± SEM, as absolute numbers or as median and interquartile range (IQR). For comparison of groups, Kruskall-Wallis-Test and post-hoc Bonferroni adjustment were used. Different superscript letters indicate significant differences between subgroups. *χ^2^-Test.

| **Table S4 Indications for open abdominal surgery** | | | | | |
| --- | --- | --- | --- | --- | --- |
|  | | **Control** | **T2D** | **Sepsis** | **p-value** |
| **Liver resection** | |  |  |  | 0.432* |
|  | benign liver disease | **4** | **1** | **-** |  |
|  | primary hepatic or biliary malignancy | **1** | **6** | **-** |  |
|  | secondary hepatic malignancy | **6** | **5** | **-** |  |
| **Gastrointestinal (GI) surgery** | |  |  |  |  |
|  | benign GI disease | **2** | **3** | **-** |  |
|  | GI malignancy | **3** | **3** | **-** |  |
| **Pancreatic surgery** | |  |  |  |  |
|  | Pancreatic carcinoma | **1** | **2** | **-** |  |
| **Septic operations** | |  |  |  | - |
|  | Insufficiency of GI anastomosis | **-** | **-** | **2** |  |
|  | GI perforation | **-** | **-** | **3** |  |
|  | GI ischaemia | **-** | **-** | **1** |  |
|  | Insufficiency of pancreatic anastomosis | **-** | **-** | **3** |  |
|  | Necrotizing pancreatitis | **-** | **-** | **1** |  |
|  | Abscess | **-** | **-** | **1** |  |
|  | Fistula | **-** | **-** | **1** |  |

*χ²-Test

| **Table S5 Correlation of chemerin with clinical and paraclinical parameters in the first study cohort** | | | | | | | | | |
| --- | --- | --- | --- | --- | --- | --- | --- | --- | --- |
|  | **Control** | | |  | **T2D** | |  | **Sepsis** | |
|  | r | p |  | | r | p |  | r | p |
| Age | 0.020 | 0.940 |  | | 0.017 | 0.942 |  | -0.454 | 0.103 |
| BMI | 0.231 | 0.372 |  | | 0.407 | 0.075 |  | -0.083 | 0.777 |
| CRP | -0.119 | 0.649 |  | | 0.327 | 0.160 |  | -0.049 | 0.867 |
| IL6 | -0.031 | 0.906 |  | | 0.206 | 0.397 |  | -0.193 | 0.507 |
| Leukocytes | 0.259 | 0.316 |  | | 0.186 | 0.433 |  | 0.628 | **0.016** |
| Thrombocytes | 0.363 | 0.152 |  | | 0.415 | 0.069 |  | 0.585 | **0.028** |
| HbA_1c_ | 0.016 | 0.951 |  | | 0.341 | 0.153 |  | 0.065 | 0.825 |
| HOMA-IR | 0.061 | 0.815 |  | | -0.233 | 0.337 |  | 0.193 | 0.508 |
| GFR | 0.034 | 0.898 |  | | 0.042 | 0.862 |  | 0.086 | 0.852 |
| Albumin | -0.365 | 0.149 |  | | -0.545 | **0.013** |  | -0.463 | 0.177 |
| Bilirubin | -0.250 | 0.332 |  | | -0.307 | 0.188 |  | -0.335 | 0.264 |
| ALAT | 0.149 | 0.567 |  | | -0.305 | 0.191 |  | -0.391 | 0.235 |
| γGT | 0.443 | 0.075 |  | | -0.210 | 0.374 |  | 0.041 | 0.904 |
| Quick | 0.261 | 0.311 |  | | -0.115 | 0.629 |  | 0.031 | 0.920 |

BMI, Body Mass Index; CRP, C-reactive protein; IL6, interleukin 6; HbA1c, Haemoglobin A1c; HOMA-IR, Homeostasis Model Assessment: GFR, glomerular filtration rate; ALAT, alanine aminotransferase; γGT, gamma glutamyltransferase

| **Table S6 Correlation of chemerin with clinical and paraclinical parameters in second cohort** | | |
| --- | --- | --- |
|  | **Control** | |
|  | r | p |
| Age | 0.072 | 0.681 |
| BMI | 0.247 | 0.158 |
| CRP | 0.127 | 0.455 |
| Leukocytes | -0.012 | 0.945 |
| Thrombocytes | 0.056 | 0.744 |
| Glucose | 0.253 | 0.131 |
| GFR | -0.255 | 0.133 |
| Urea | 0.299 | 0.073 |
| Bilirubin | -0.080 | 0.639 |
| Quick | 0.021 | 0.900 |

BMI, Body Mass Index; CRP, C-reactive protein; IL6, interleukin 6; HbA1c, Haemoglobin A1c; HOMA-IR, Homeostasis Model Assessment: GFR, glomerular filtration rate; ALAT, alanine aminotransferase; γGT, gamma glutamyltransferase
