# Supplementary figures and images for: Chemerin in peritoneal sepsis and its associations with glucose metabolism and prognosis: a translational cross-sectional study
Source: Crit Care. 2016 Feb 12;20:39. doi: 10.1186/s13054-016-1209-5 (PMC4751629; doi:10.1186/s13054-016-1209-5)

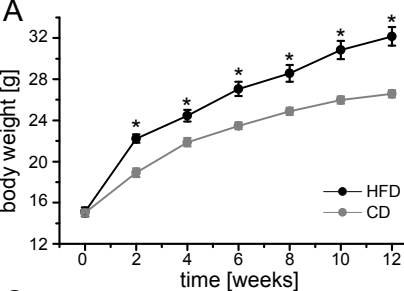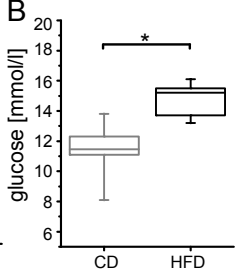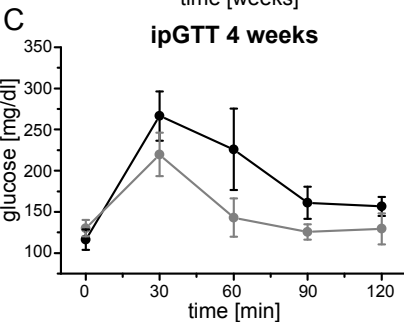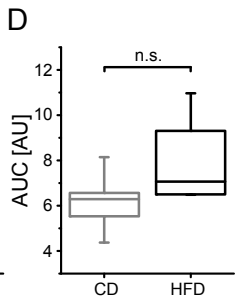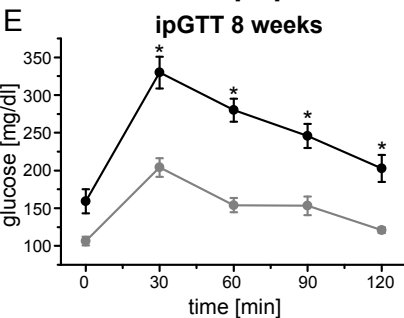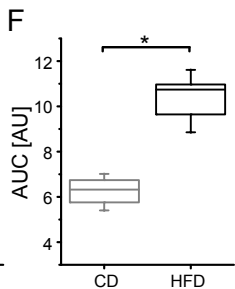

Supplement: Additional file 2: — Figure S1. Characterisation of the murine HFD model. a Development of body weight over 12 weeks of high-fat feeding. HFD mice gained significantly more body weight (n = 15/group). b Baseline plasma glucose levels were significantly higher in HFD-fed mice after 12 weeks compared with CD-fed mice (p = 0.01, n = 6). c Intra-peritoneal glucose tolerance test (ipGTT) showed no significant difference in glucose tolerance after 4 weeks of HFD (n = 4 or 5). d Area under the curve (AUC) was not significantly different for ipGTT between HFD- and CD-fed mice after 4 weeks of feeding (p = 0.286; n = 4 or 5). e HFD-fed mice had decreased glucose tolerance in ipGTT after 8 weeks of feeding (p = 0.04; n = 5). f Area under the curve (AUC) was significantly higher in HFD-fed mice compared with CD-fed mice after 8 weeks of feeding (p = 0.008, n = 5). (PDF 47 kb) [file 13054_2016_1209_MOESM2_ESM.pdf]

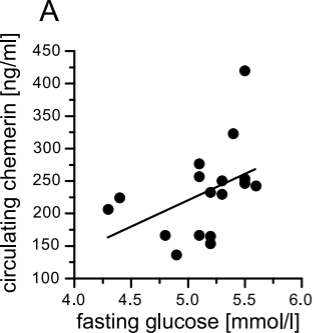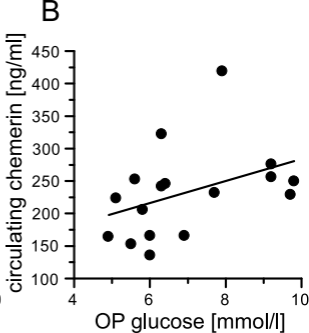

Supplement: Additional file 3: — Figure S2. Correlation of fasting and intra-operative glucose levels with circulating chemerin in controls. a Correlation of circulating chemerin levels with fasting glucose levels (r = 0.216, p = 0.034, n = 17). b Correlation of circulating chemerin levels with OP glucose (r = 0.549, p = 0.023, n = 17). (PDF 44 kb) [file 13054_2016_1209_MOESM3_ESM.pdf]

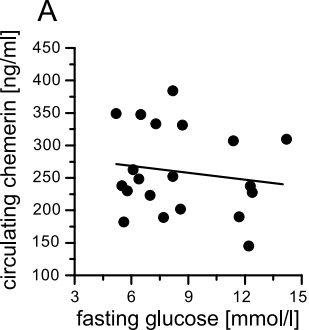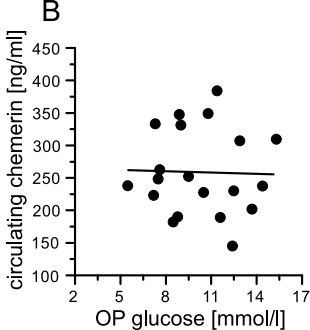

Supplement: Additional file 4: — Figure S3. Correlation of fasting and intra-operative glucose levels with circulating chemerin in T2D. a Correlation of circulating chemerin levels with fasting glucose levels (r = −0.149, p = 0.53, n = 21). b Correlation of circulating chemerin levels with OP glucose (r = −0.025, p = 0.92, n = 21). (PDF 44 kb) [file 13054_2016_1209_MOESM4_ESM.pdf]
